# *Drosophila* Porin/VDAC Affects Mitochondrial Morphology

**DOI:** 10.1371/journal.pone.0013151

**Published:** 2010-10-07

**Authors:** Jeehye Park, Yongsung Kim, Sekyu Choi, Hyongjong Koh, Sang-Hee Lee, Jin-Man Kim, Jongkyeong Chung

**Affiliations:** 1 National Creative Research Initiatives Center for Energy Homeostasis Regulation, Seoul National University, Seoul, Korea; 2 School of Biological Sciences, Seoul National University, Seoul, Korea; 3 Department of Biological Sciences, Korea Advanced Institute of Science and Technology, Daejeon, Korea; 4 Department of Pharmacology, Dong-A University College of Medicine, Busan, Korea; 5 Department of Pathology, Chungnam National University School of Medicine, Daejeon, Korea; 6 Institute of Molecular Biology and Genetics, Seoul National University, Seoul, Korea; VIB, Belgium

## Abstract

Voltage-dependent anion channel (VDAC) has been suggested to be a mediator of mitochondrial-dependent cell death induced by Ca^2+^ overload, oxidative stress and Bax-Bid activation. To confirm this hypothesis *in vivo*, we generated and characterized *Drosophila VDAC (porin)* mutants and found that Porin is not required for mitochondrial apoptosis, which is consistent with the previous mouse studies. We also reported a novel physiological role of Porin. Loss of *porin* resulted in locomotive defects and male sterility. Intriguingly, *porin* mutants exhibited elongated mitochondria in indirect flight muscle, whereas Porin overexpression produced fragmented mitochondria. Through genetic analysis with the components of mitochondrial fission and fusion, we found that the elongated mitochondria phenotype in *porin* mutants were suppressed by increased mitochondrial fission, but enhanced by increased mitochondrial fusion. Furthermore, increased mitochondrial fission by Drp1 expression suppressed the flight defects in the *porin* mutants. Collectively, our study showed that loss of *Drosophila* Porin results in mitochondrial morphological defects and suggested that the defective mitochondrial function by Porin deficiency affects the mitochondrial remodeling process.

## Introduction

Mitochondria undergo mitochondrial membrane permeability transition (MPT) after opening of a channel called mitochondrial permeability transition pore (PTP) when cells succumb to apoptosis [1]. PTP is a site for cytochrome c release, which then leads to caspase activation and cell death. PTP is minimally composed of three proteins, VDAC (in the outer membrane), the adenine nucleotide translocase (ANT, in the inner membrane) and cyclophilin D (CypD, in the matrix). These PTP components have been initially proposed to be critically involved in mitochondrial cell death induced by Ca^2+^ overload, oxidative stress and Bax-Bid activation. However, recent studies have demonstrated that VDAC and ANT are not essential for MPT in cell death but only cyclophilin D required [2]–[4]. Therefore, the physiological role of VDAC and ANT remains questionable.

There are three *VDAC* isoforms in mammals, but in *Drosophila* four isoforms have been identified homologous to *VDAC* [5]–[6]. Among them, *porin* (CG6647) and *porin2* (CG17137) show a higher similarity to mammalian *VDAC* (*porin*, about 58% identity; *porin2*, about 34% identity), whereas the two others, CG17139 and CG17149, show about 24% identity and 21% identity, respectively. Previous *Drosophila* Porin protein studies have demonstrated that Porin and Porin2 have similar functions because both proteins rescued the conditional lethal phenotype of yeast *VDAC* mutants [5]. Interestingly, gene expression studies have shown that Porin is ubiquitous in all body segments, whereas Porin2 is detected prominently in sperm tissue [6]–[8]. Therefore, Porin may play a general role in many different tissues.

Mitochondria constantly move to specific subcellular locations where high energy is demanded by undergoing morphological changes through mitochondrial fusion and fission [9]. These processes have been first identified in yeast, and several genes have been found conserved in mammals; *mitofusin 1* (*mfn1*) and *mitofusion 2* (*mfn2*), both encoding GTPases required for the outer membrane fusion, *optic atrophy 1* (*opa1*), a GTPase for the inner membrane fusion, and *dynamin-related protein 1* (*drp1*), a GTPase for mitochondrial fission. In *Drosophila*, the two homologs of *mfn* are *fuzzy onion* (*fzo*) and *mitochondrial assembly regulatory factor* (*marf*). *fzo* mutants show defects in mitochondrial fusion during spermatogenesis and knockdown of Marf induces mitochondrial fission in indirect flight muscle [10]–[11]. Recent studies have also demonstrated that *Drosophila opa1* and *drp1* mutants show defects in mitochondrial morphology [12]–[13].

To investigate the physiological role of Porin, we generated *porin* null mutants and characterized them in *Drosophila*. Our studies showed that loss of *porin* results in elongated mitochondria and Porin overexpression induces small mitochondria. Through genetic interaction analysis between Porin and mitochondrial fusion and fission regulators, we revealed that loss of *porin* leads to defective mitochondrial remodeling processes in *Drosophila*.

## Results

**Figure 1:**
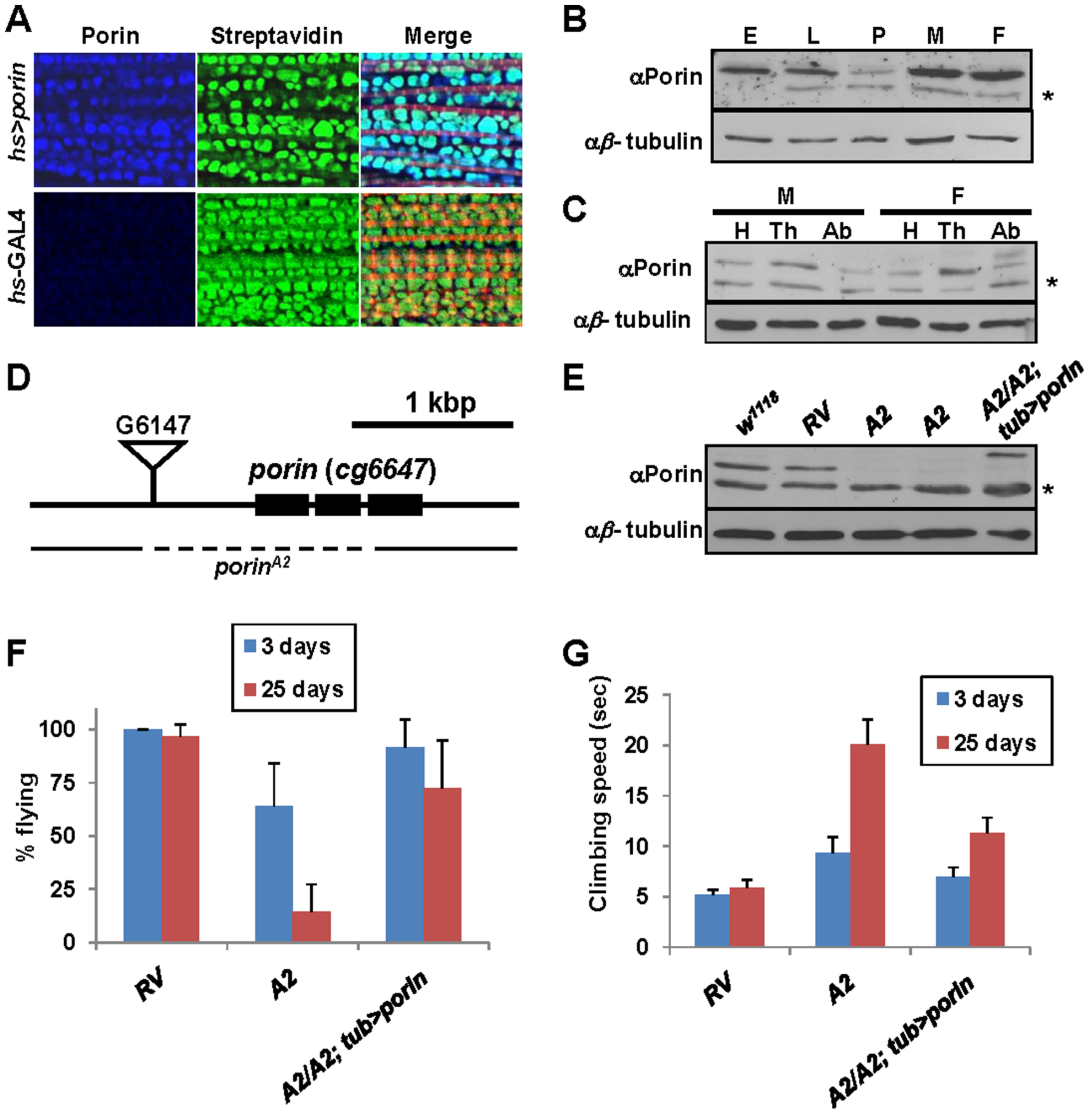
Characterization of *Drosophila porin* loss-of-function mutants. (A) Mitochondrial localization of Porin. Subcellular localization of Flag-tagged Porin (blue) in indirect flight muscle. Streptavidin (green), and phalloidin (red) were used to mark mitochondria, and muscle fiber respectively. (B) Developmental expression pattern of *Drosophila* Porin. Immunoblot analysis of Porin from different developmental stages of wild type flies. Anti-Porin rabbit antibody binds specifically to the wild type samples, but not to *porin* null mutants (shown in Fig. 1E). E, embryo; L, larva; P, pupa; M, male; F, female. n = 3. * indicates a non-specific band. (C) Porin expression in head (H), thorax (Th) and abdomen (Ab) of adult flies of each sex (M, male; F, female). n = 3. (D) Genomic region of *porin* locus. The exons of *porin* gene are depicted as black boxes. The inverted triangle in front of *porin* gene is the P element used for imprecise excision. The deleted region for *porin^A2^* allele is indicated. (E) Verification of *Drosophila porin* mutants through immunoblot analysis. Porin protein is absent in *porin* null mutants (*A2*). (F) Comparison of the flight ability. n = 10. S.D. for three independent experiments. (G) Comparison of the climbing rate. n = 10. S.D. for three independent experiments.

We first examined whether Porin protein localizes to the mitochondria. Through immunohistochemistry with indirect flight muscles of flies, Porin was mostly detected in the mitochondria (Figure 1A). We then examined the developmental and spatial expression patterns for Porin by performing immunoblot analysis. Porin protein was detected in all developmental stages (Figure 1B). Its expression in adults was ubiquitous throughout all segments, head, thorax and abdomen (Figure 1C). These results are consistent with the previous studies [6].

To investigate the *in vivo* function of Porin in *Drosophila*, we determined to generate *porin* loss-of-function mutants by mobilizing the P-element G6147 inserted near the *porin* locus (Figure 1D). Through P-element imprecise excision, a *porin*-deleted allele was isolated and named *porin^A2^* (*A2*, Figure 1D). This allele has a 1,381bp-deletion, removing the start codon of translation and the first two exons, confirmed by genomic DNA-PCR analysis and DNA sequencing (data not shown). We also generated *porin* revertant line (*porin^RV^*, *RV*) to use as a control. To further assess whether Porin expression is completely eliminated in the *porin* homozygous mutants, immunoblot analysis was performed and confirmed that the allele is null for *porin* (Figure 1E).

The homozygous *porin* mutants were viable and the adults seemed normal after eclosion, which is consistent with previous studies [14]. However, they started to show defects in their behaviors such as slow climbing ability against geotaxis and flying disability at the age of 20 days and then died within 30 days (Figure 1F, 1G, and Figure S1, respectively).

**Figure 2:**
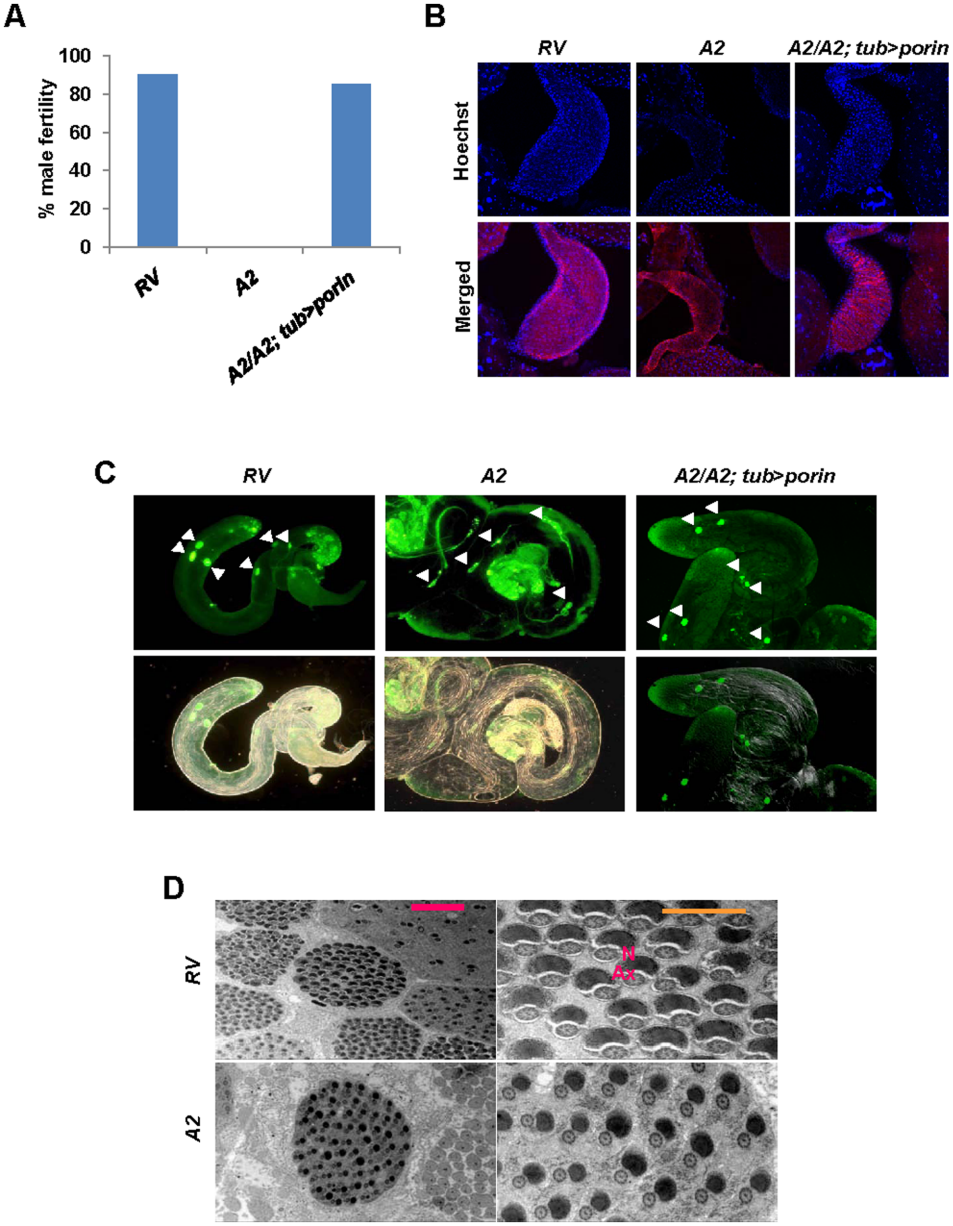
Sperm individualization defects in *porin* mutants. (A) Comparison of fertility of males of indicated genotypes. n = 30. (B) Mature sperms determined by staining Hoechst33258 in the pouch *vas deferens* in 3-day-old male testis. Co-immunostaining with phalloidin (red) was performed to show the overall morphology of the pouch. *porin* mutants do not produce mature sperms whereas the control flies show linear morphology of nucleus of mature sperms. (C) Defects in the morphology of cystic bulges (CBs, white arrows) and waste bags (WBs) in *porin* mutants determined by acridine orange (AO) staining of testes. The control and the ectopic *porin* expression in *porin* mutants show intact CBs/WBs, but *porin* mutants show long-tailed signals. (D) TEM analysis of syncytial cyst of 64-spermatids. Red scale bar, 2 µm; orange scale bar, 1 µm. Ax, axoneme; N, nebenkern.

Homozygous female *porin* mutants were fertile, but male mutants were sterile (data not shown and Figure 2A). We observed the testis of *porin* mutants (*A2*), and the revertants (*RV*) by co-staining with Hoechst to mark sperms and phalloidin for the overall organ. As a result, we could not detect any mature sperms in the pouch of *porin* mutants called *vas deferens*, which is connected to the anterior ejaculatory duct, and this phenotype was rescued by ectopic *porin* expression (Figure 2B). To further investigate the cause of sterility of *porin* mutants, we assessed each stage of spermatogenesis development. In *Drosophila* testis, stem cells on the tip of the testis undergo differentiation and then go into rounds of mitotic division followed by meiosis, thereby producing syncytial cysts of 64 spermatids [15]. All these early developmental events seemed normal in *porin* mutants (Figure S2A). Therefore, we looked for defects by further observing the stages after post-meiosis; the interconnected 64 spermatids must undergo axoneme assembly, spermatid elongation, individualization, and coiling [15]. Through phase-contrast microscopy, we were able to find elongated spermatids (data not shown). However, immunostaining analysis showed that *porin* mutants have defects in spermatid individualization (Figures 2C and S2B). To understand the phenotype, we performed transmission electron microscopy (TEM) analysis with the spermatids. Interestingly, the mitochondria (N, nebenkern) in the wild type were fully condensed near axnome (Ax), but the mitochondria in the mutants were less condensed (Figure 2D). These results suggested that the defects in sperm individualization of *porin* mutants are related to mitochondrial dysfunction.

**Figure 3:**
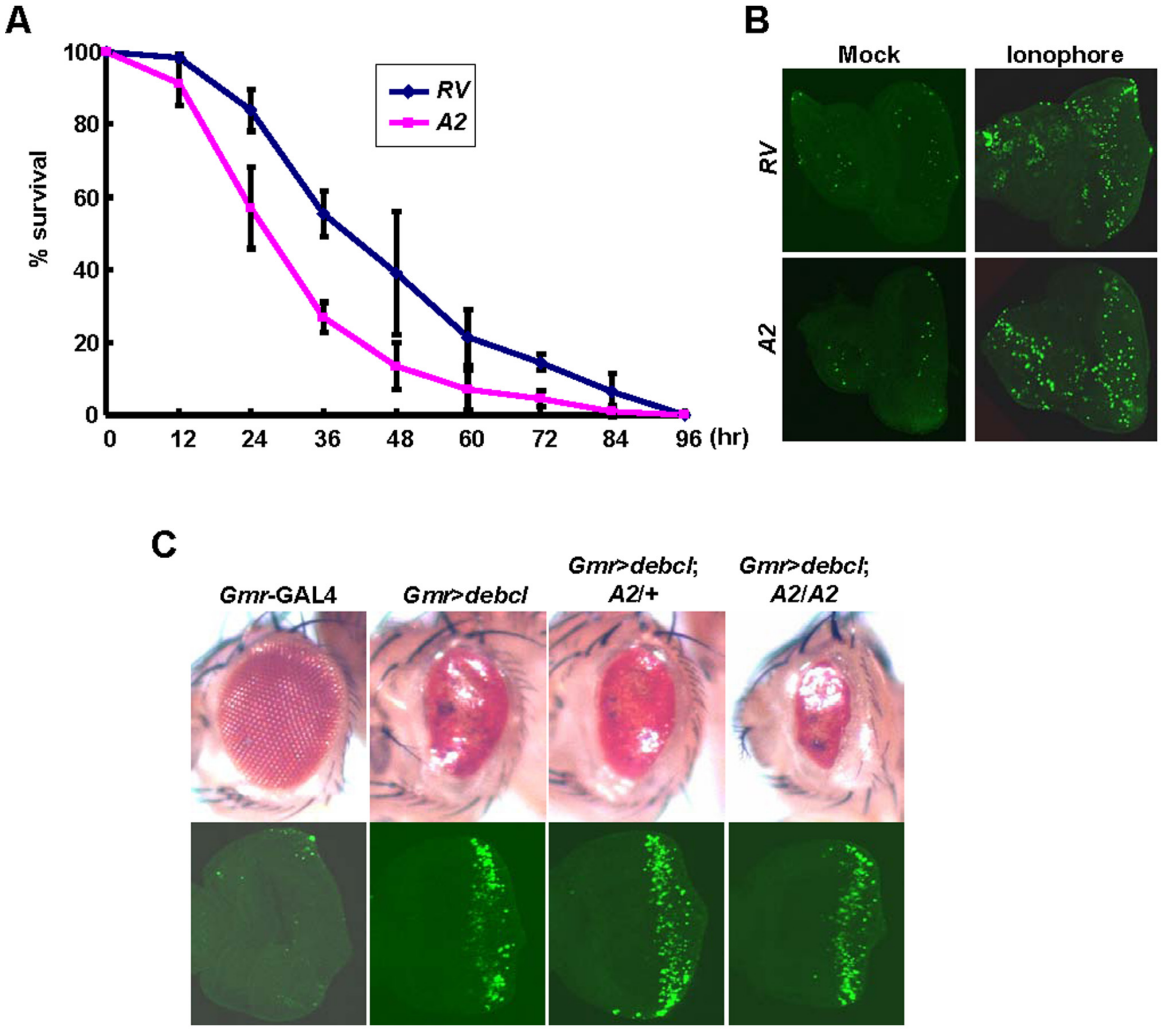
*Porin* is not resistant to oxidative stress, ionophore or Bax-induced cell death. (A) Graph of % survival of 20mM paraquat-fed flies counted every 12 hr. n = 100. S.D. for three independent experiments. (B) TUNEL staining of the eye discs of indicated genotypes after incubation with ionophore. At least three independent experiments were performed. (C) Microscopy images of the eyes (*upper* panels) and TUNEL staining of the eye discs (*lower* panels) of indicated genotypes. Details of all indicated genotypes are described in Supporting Information File S1.

Since Porin was previously considered as an essential factor for MPT in cell death, we first tested this hypothesis using our *Drosophila porin* mutants. If Porin plays a role in promoting cell death induced by oxidative stress, Ca^2+^ overload and Bax activation, *porin* null mutants should be resistant to apoptosis following these toxic treatments. However, we found that Porin has no role in this process *in vivo* as we have obtained the following results. We investigated the effects of oxidative stress using paraquat on the survival of *porin* mutants. Flies were reared in 20 mM paraquat agar-contained vial and their survival was checked every 12 hrs. We found that *porin* mutants (*A2*) die at a rate similar to that of the revertants (*RV*) or a little faster (Figure 3A), suggesting that *porin* mutants are not resistant to oxidative stress. We further investigated whether *porin* null mutants are resistant to ionophore-induced cell death. The eye discs of *porin* mutants were incubated in A23187-containing M3 media for 4 hrs and observed for apoptosis by performing TUNEL assay. We found that the eye discs of *porin* mutants (*A2*) showed apoptosis as much as that of the revertants (*RV*) (Figure 3B), suggesting that Porin is not required for cell death mechanism induced by this stimulation. It has been also proposed that pro-apoptotic members of the Bcl-2 protein family such as Bax could promote MPT-induced cell death by binding to VDAC [16]. Overexpression of Debcl (*Drosophila* Bax) induced smaller eye phenotypes (Figure 3C). However, this phenotype was not rescued under heterozygous nor homozygous *porin* null background (Figure 3C), suggesting that Porin is not required for Bax-induced cell death. We also performed TUNEL assay to assess apoptosis in the eye discs of the mutants and found that apoptosis induced by Debcl overexpression was not inhibited by *porin* deletions (Figure 3C). Collectively, our results demonstrated that Porin is dispensable for cell death induced by oxidative stress, Ca^2+^ overload or Bax activation. These results are fully consistent with the previous *VDAC* mouse studies [2].

**Figure 4:**
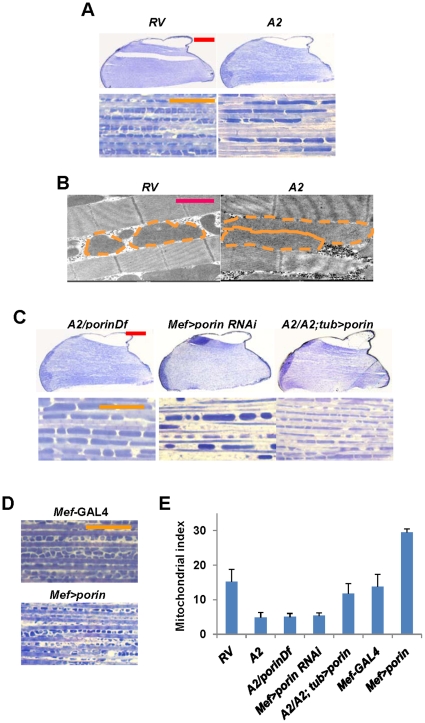
*Porin* mutants show defects in mitochondrial morphology. (A) Longitudinally sectioned thorax images stained with toluidine blue. Red scale bar, 200 µm; orange scale bar, 20 µm. (B) TEM analysis of mitochondria in the indirect flight muscle of 3-day-old males. Red scale bar, 1 µm. Orange dotted line was drawn to show the mitochondrial size clearly. (C and D) Longitudinally sectioned thorax images stained with toluidine blue. Details of all indicated genotypes are described in Supporting Information File S1. (E) Quantification of the number of mitochondria within 50µm between two muscle fibers (mitochondrial index) shown in (A), (C), and (D). n>10.

**Figure 5:**
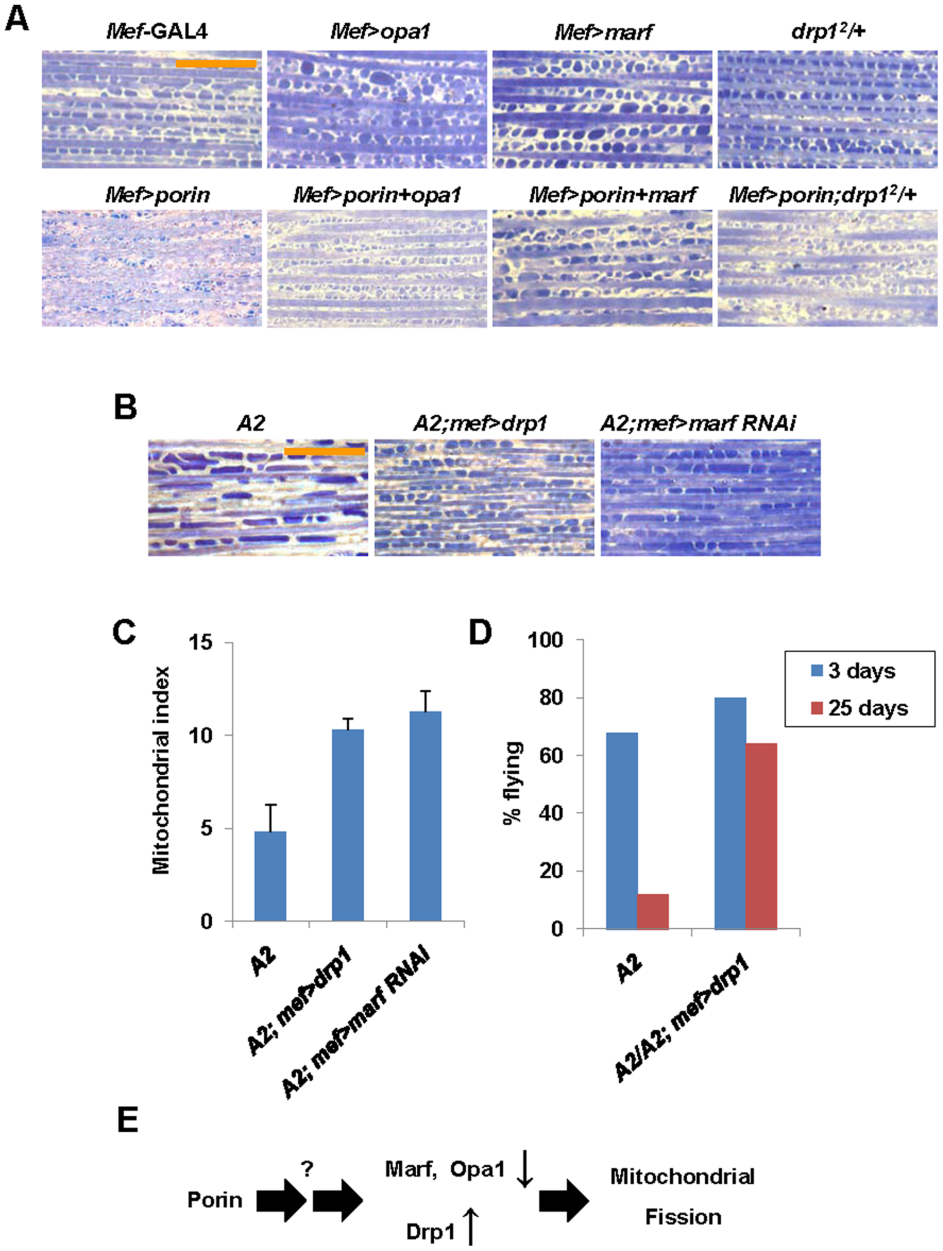
Genetic interaction analyses between Porin and the components involved in mitochondrial remodeling process. (A and B) Longitudinally sectioned thorax images stained with toluidine blue. Orange scale bar, 20 µm. Details of all indicated genotypes are described in Supporting Information File S1. (C) Quantification of the number of mitochondria within 50µm between two muscle fibers (mitochondrial index) shown in (B). n>10. (D) Comparison of the flight ability shown in (B). n = 50. S.D. for three independent experiments. (E) A schematic illustration showing the function of Porin in mitochondrial fusion and fission proteins.

To reveal a novel role of Porin *in vivo*, we further characterized *porin* mutants. Because mitochondrial dysfunction was evident in the sperm of *porin* mutants, we assessed the mitochondrial morphology in another tissue, indirect flight muscle, where mitochondria could be easily observed. Through muscle sections of the thoraces embedded in Spurr's resin followed by coloring with toluidine blue, dark blue mitochondria were visible between the muscle fiber bands in light blue color in control flies (Figures 4A and 4E). However, it was surprising to see that the sections of *porin* mutants showed elongated tube-like mitochondria between the muscle fibers (Figures 4A and 4E). This result was further supported by examining the indirect flight muscle of *porin* mutants using TEM analysis. Indeed, *porin* mutants showed electron-dense elongated mitochondria in between the muscle fibers (Figure 4B). Moreover, *porin^A2^* (*A2*) allele crossed to a *porin*-deficiency line (*porinDf*) showed the identical phenotype (Figures 4C and 4E). We further confirmed this phenotype by generating a *porin* RNAi line, and overexpression of *porin* RNAi by muscle specific *mef*-GAL4 driver led to similar phenotypes (Figures 4C and 4E). In addition, expression of Porin under *tubulin*-GAL4 (*tub*-GAL4) significantly rescued the *porin* mutant phenotype (Figures 4C and 4E), suggesting that the defects in mitochondrial morphology were caused by loss of *porin* gene. We then investigated whether overexpression of Porin produces the opposite phenotype. As expected, we found smaller mitochondria in between the muscle fibers in Porin-overexpressed indirect flight muscles (Figures 4D, 4E, and 5A). Collectively, these results suggest that Porin is important for maintaining mitochondrial morphology.

Because the changes in the level of Porin protein affect mitochondrial morphology in the indirect flight muscle, we thought that Porin might be involved in mitochondrial remodeling process. To further provide evidence to support our hypothesis, we conducted genetic interaction analysis between Porin and mitochondrial fusion and fission proteins. First, we tested whether the mitochondrial fission phenotype induced by Porin overexpression is altered by mitochondrial fusion and fission molecules. We therefore obtained several loss-of-function mutants or deficiency lines of *drp1*, *opa1* and *marf* and generated UAS-*drp1*, UAS-*opa1* and UAS-*marf* transgenic lines.

First, we tested the genetic interaction between Porin and Opa1. A heterozygous mutation of *opa1* induced smaller mitochondria than those of wild type (Figures S3A and S4A), and overexpression of Opa1 results in larger mitochondria (Figures 5A and S4A). Coexpression of Opa1 significantly rescued the Porin overexpression phenotype (Figures 5A and S4A), but a heterozygotic mutation of *opa1* further enhanced the phenotype induced by Porin expression (Figures S3A and S4A). Similarly, Marf overexpression resulted in a similar phenotype to that of Opa1 overexpression (Figures 5A and S4B), whereas flies with *marf* heterozygotic background showed mitochondrial size similar to, albeit smaller than, those of wild type flies (Figures S3B and S4B). The fragmented mitochondrial phenotype induced by Porin overexpression was suppressed by Marf overexpression (Figures 5A and S4B), but this phenotype was enhanced in a *marf* heterozygotic background (Figures S3B and S4B). In addition, coexpression of Drp1 and Porin showed fragmented mitochondria, whereas overexpression of Drp1 alone showed an almost normal mitochondrial morphology (Figures S3C and S4C). Overexpression of Porin with a heterozygotic mutation of *drp1* produced smaller mitochondria or almost completely abolished the mitochondria (Figures 5A and S4C).

To further prove the role of Porin in mitochondria, we examined whether elongated mitochondria in *porin* null mutants are altered by increasing the level of mitochondrial fission genes. Expectedly, homozygous *porin^A2^* (*A2*) phenotype was strongly suppressed by Drp1 overexpression or Marf knockdown (Figures 5B and 5C) and the flight disability in *porin* mutants was rescued by Drp1 overexpression (Figure 5D). These results suggested that Porin affects mitochondrial remodeling, and that the defective mitochondrial function by Porin deficiency appears to promote mitochondrial fusion or inhibit mitochondrial fission.

## Discussion

Mitochondria are dynamic organelles, which constantly fuse and divide and move to specific subcellular locations where energy demands are high [17]. Controlling mitochondrial remodeling process is not only important for maintaining mitochondrial morphology but also for mitochondrial functions, which may determine the cellular activity and survival [17]. However, there is not much known about how this event is regulated or is related to apoptosis, especially MPT.

Recently, researchers studying the function of PTP components have questioned about the interaction between mitochondrial fusion and fission and the MPT components [18]. To address the question, we generated VDAC, one of the core components of PTP, null flies, but found that Porin is dispensable for oxidative stress-, Ca^2+^ overload- or Bax activation-induced cell death (Figure 3). Rather, we now suggest a novel role of VDAC in mitochondrial remodeling process. Overexpression of *Drosophila* Porin induced mitochondrial fission, whereas its loss-of-function resulted in mitochondrial fusion in muscle tissues (Figure 4). Furthermore, *Drosophila* Porin showed strong genetic interactions with known mitochondrial fusion and fission proteins. Particularly with expression of Drp1, not only we found a significant rescue of the mitochondrial morphology in the indirect flight muscle of *porin* mutants but also in their flight ability (Figures 5B and 5D). Similar mitochondrial phenotypes were also shown in the previous studies with *VDAC1* null mice [3], which further support our results. Most recently, another group has published a paper on the fly mutant of the same gene, but generated differently from a different P-element mutant origin, and their mutants have the same phenotype as ours [19].

We also wanted to know whether genetic interaction between Porin and mitochondrial fusion/fission proteins is also conserved in different tissues, so we observed the rescue effect of mitochondrial fusion/fission genes on male infertility of *porin* mutants (data not shown). Interestingly, an increase in mitochondrial fission by overexpression of Drp1 or *marf* RNAi could not rescue the phenotype of *porin* mutants. We thought that it was because the spermatogenesis is highly complex event, so only inducing mitochondrial fission could not restore the defects or the timing of ectopic gene expression during spermatogenesis was not appropriate. Further studies are required to determine the etiology of this tissue-specific phenotype.

In addition, another PTP component ANT, located in the mitochondrial inner membrane, has been also demonstrated as a nonessential component for MPT [4]. We found that *SesB*, *Drosophila ANT*, genetically interacted with Porin in promoting mitochondrial fission because small mitochondria induced by Porin overexpression were markedly rescued by a heterozygotic mutation of *sesB* in fly muscle tissues (data not shown). These results strongly suggest that the functions of PTP or, at least, some components of PTP including VDAC and ANT control mitochondrial remodeling processes either directly or indirectly.

Although we showed that *Drosophila* Porin can be genetically involved in mitochondrial remodeling process, we cannot exclude the possibility that the morphological changes of mitochondria in *porin* mutants is an indirect effect or a mere consequence of unhealthy mitochondria induced by loss of Porin functions. Further genetic and biochemical analysis are required for detailed understanding of how PTP components affect mitochondrial remodeling processes.

In the present study, our *Drosophila* genetic data revealed that Porin may be related with mitochondrial remodeling process. We believe that our finding will further enhance the understanding of the molecular mechanism of mitochondrial remodeling and the physiological role of VDAC/Porin and PTP.

## Materials and Methods

### Fly stocks

We have generated UAS-*drp1* (C-terminally HA-tagged), UAS-*opa1* (C-terminally Flag-tagged) and UAS-*marf* (C-terminally Flag-tagged) transgenic lines, and their expression was confirmed by immunoblot analyses. UAS-*debcl* [20], *opa1^EP^* (*opa1^p{EPgy2}CG8479^*) [12] and *drp1^2^* [13] line were kindly provided by S. Kumar, A. Mcquibban, and H. J. Bellen, respectively. *Mef*-GAL4 was kindly provided by E. N. Olson [21]. *Hs*-GAL4, *tub*-GAL4, *gmr*-GAL4, *porinDf* (Df(2L)BSC143, BL#7142) and *marfDf* (Df(1)dx81, BL#5281) lines were obtained from the Bloomington Stock Centre (Bloomington, IN). *marf* RNAi line was obtained from the Vienna Drosophila Resource Centre.

### Generation of porin loss-of-function and overexpression flies

From the GenExel library, we isolated a P-element insertion line in the second exon of the gene. *porin^A2^* allele was generated through imprecise excision of the P-element and was confirmed to be a loss-of-function mutant for *porin*. We also generated a revertant (*RV*) allele (*porin^RV^*) by precise excision. For generation of UAS-*porin* flies, *Drosophila porin* EST (clone #GH11331) was obtained from DGRC. The entire open reading frame (ORF) was subcloned into N-terminally Flag-tagged pUAST vector. *porin* RNAi line was generated through PCR using this primer pair: 5′-GCGGAATTCCTGGATGTGGGTGTACAGC-3′ and 5′-CGCGAATTCCAGCTCCAGACCCACACC-3′ and then cloned into pSymp vector. These generated constructs were subjected to DNA sequencing for validation and then microinjected into *w^1118^* embryos for generation of transgenic flies.

### Fly behavioral assays

Flight ability and climbing assays were performed as previously described [22] with 3- or 25-day-old males (n>30). Fly survival was examined as previously described [22].

### Male fertility test

Individual 3-day-old males of *RV*, *A2*, *and A2/A2*; *tub>porin* were allowed to mate with two virgin *w^1118^* females. The fully mated females were allowed to lay eggs for the same period (∼1 day) on the standard medium. After 15 days, the number of adult progenies was counted for fertility.

### Mitochondrial staining in the thorax muscle

Fly thoraces were fixed with 4% paraformaldehyde for 10 min at 4°C and then were cut in half by dissecting vertically along the bristles in the middle of the thorax. They were then blocked in PBS-T with 2% BSA for 1 hr at room temperature and were incubated with Alexa 488-conjugated streptavidin (Invitrogen) for mitochondria visualization overnight at 4°C. After several rounds of washing with PBS-T, they were dehydrated in serial dilutions of ethanol and then were added with methylsalicylate (Sigma). They were mounted on a hollow glass slide (Matsunami, Japan) due to the thorax volume.

### Paraquat-sensitivity assay

This assay was performed as previously described [23] but with some modifications. 1–3 day old flies were reared in 20 mM paraquat agar-contained vial and their survival was checked every 12 hrs.

### Apoptosis assay by ionophore treatment

This experiment was conducted as previously described [24] with some modifications. The eye discs were incubated in A23187-containing M3 media for 4 hrs and then observed for apoptosis by performing TUNEL assay.

### Immunostaining and TUNEL assay

Indirect flight muscle samples were stained with Mitotracker for 30 min and fixed with 4% paraformaldehyde and blocked in TBST with 2% BSA. For TUNEL assay, apoptosis in the eye discs of third instar larvae was detected using the *in situ* cell death detection kit (Roche).

### Generation of porin antibody

The polyclonal antibody to *Drosophila* porin was generated in rabbit by injecting porin peptide (Anygen, Korea) and further purified.

### Immunohistochemistry

To observe the sperms at the stage of spermatid individualization, immunohistochemistry was conducted as previous described [25].

### Mitochondrial index

The number of mitochondria within 50 µm between two fibers in thorax from a single image frame were grouped and averaged; means from multiple thoraces were averaged to obtain a population mean and S.D.

## Supporting Information

File S1 Supplementary Genotypes
(0.03 MB DOC)

Figure S1 Reduced lifespan of *porin* mutants. n = 100. S.D. for three independent experiments.
(0.07 MB TIF)

Figure S2 Examination of sperm. (A) No difference in the morphology of sperms between the onion stages (right panels) or earlier stages (left panels) of control and *porin* mutants. (B) Defects in spermatid individualization of *porin* mutants determined by anti-active Drice antibody (green) and phalloidin (red). Active Drice is detectable in CBs (white arrows) and WBs (red arrows). *Porin* mutants do not show CBs or WBs.
(0.44 MB TIF)

Figure S3 Genetic interaction analysis between Porin and the components involved in mitochondrial remodeling process. (A–C) Longitudinally sectioned thorax images stained with toluidine blue. Details of all indicated genotypes are described in Supplementary Information.
(0.35 MB TIF)

Figure S4 Quantification of the thorax mitochondria phenotype. (A–C) Measurement of the number of mitochondria within 50 um distance between two thorax muscle fibers (mitochondrial index) in each genotypes. Genetic interaction analysis between *porin* and *opa1* (A), *marf* (B), and *drp1* (C). n>10.
(0.08 MB TIF)
