# Supplementary material for: Drosophila Porin/VDAC Affects Mitochondrial Morphology
Source: PLoS One. 2010 Oct 7;5(10):e13151. doi: 10.1371/journal.pone.0013151 (PMC2951900; doi:10.1371/journal.pone.0013151)
Supplement: File S1 — Supplementary Genotypes (0.03 MB DOC) [file pone.0013151.s001.doc]

**Supporting Information - Details of genotypes for Main and Supporting Figures**

Jeehye Park, Yongsung Kim, Sekyu Choi, Hyongjong Koh, Sang-Hee Lee, Jin-Man Kim & Jongkyeong Chung

**Figure 3**. *gmr*>*debcl* (*gmr*-GAL4/+; UAS-*debcl*/+), *gmr*>*debcl*; *A2*/+ (*gmr*-GAL4, *porin^A2^*/+; UAS-*debcl*/+), *gmr*>*debcl*; *A2*/*A2* (*gmr*-GAL4, *porin^A2^*/*porin^A2^*; UAS-*debcl*/+).

**Figure 4**. *A2*/*porinDf* (*porin^A2^*/*porin^Df^*), *Mef*>*porin RNAi* (*Mef*-GAL4/UAS-*porin RNAi*), *A2*/*A2*; *tub*>*porin* (*porin^A2^*/*porin^A2^*; *tub*-GAL4/UAS-*porin*), Mef>porin (*Mef*-GAL4/UAS-*porin*).

**Figure 5**. *Mef*>*porin* (*Mef*-GAL4/UAS-*porin*), *Mef*>*porin*+*opa1* (*Mef*-GAL4>UAS-*porin*/UAS-*opa1*), *Mef*>*opa1* (*Mef*-GAL4/UAS-*opa1*), *Mef*>*marf* (*Mef*-GAL4/UAS-*marf*), *Mef*>*porin*+*marf* (*Mef*-GAL4>UAS-*porin*/UAS-*marf*), *Mef*>*porin*; *drp1^2^*/+ (*drp1^2^*/+; *Mef*-GAL4>UAS-*porin*/+), *A2*/*A2*; *mef*>*drp1* (*porin^A2^*/*porin^A2^*; *mef*-GAL4/UAS-*drp1*).

**Figure S3***. Mef*>*porin*; *opa1^EP^*/+ (*opa1^EP^*/+; *Mef*-GAL4>UAS-*porin*/+), *Mef*>*porin*; *marfDf*/+ (*Mef*-GAL4>UAS-*porin*/+; *marfDf*/+), *Mef*>*drp1* (*Mef*-GAL4/UAS-*drp1*), *Mef*>*porin*+*drp1* (*Mef*-GAL4>UAS-*porin*/UAS-*drp1*).
