# Supplementary figures and images for: Drosophila Porin/VDAC Affects Mitochondrial Morphology
Source: PLoS One. 2010 Oct 7;5(10):e13151. doi: 10.1371/journal.pone.0013151 (PMC2951900; doi:10.1371/journal.pone.0013151)

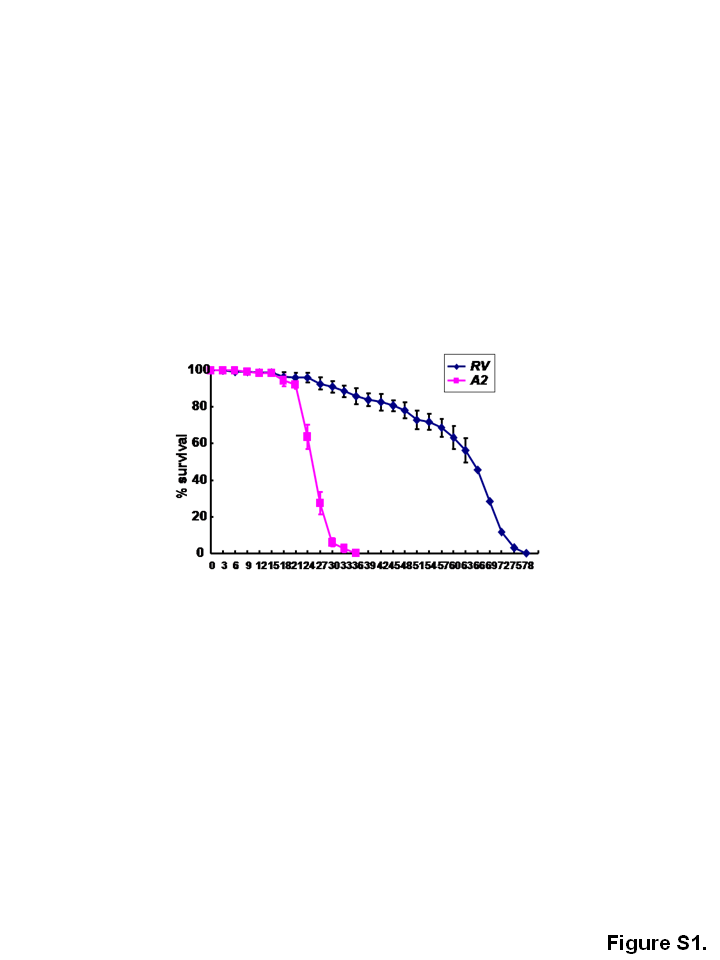

Supplement: Figure S1 — Reduced lifespan of porin mutants. n = 100. S.D. for three independent experiments. (0.07 MB TIF) [file pone.0013151.s002.tif]

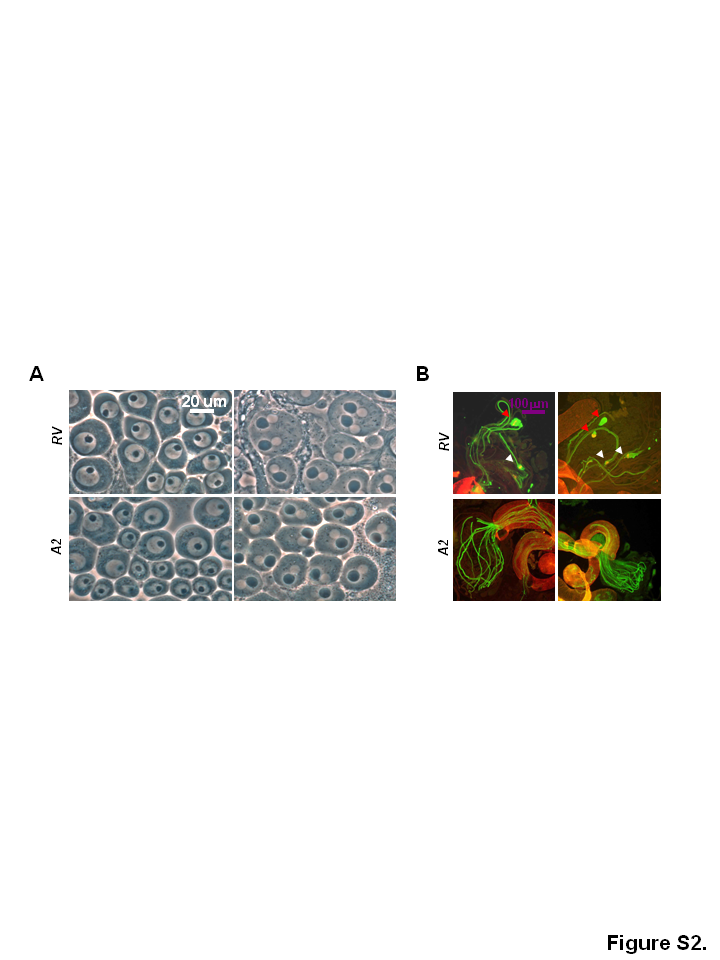

Supplement: Figure S2 — Examination of sperm. (A) No difference in the morphology of sperms between the onion stages (right panels) or earlier stages (left panels) of control and porin mutants. (B) Defects in spermatid individualization of porin mutants determined by anti-active Drice antibody (green) and phalloidin (red). Active Drice is detectable in CBs (white arrows) and WBs (red arrows). Porin mutants do not show CBs or WBs. (0.44 MB TIF) [file pone.0013151.s003.tif]

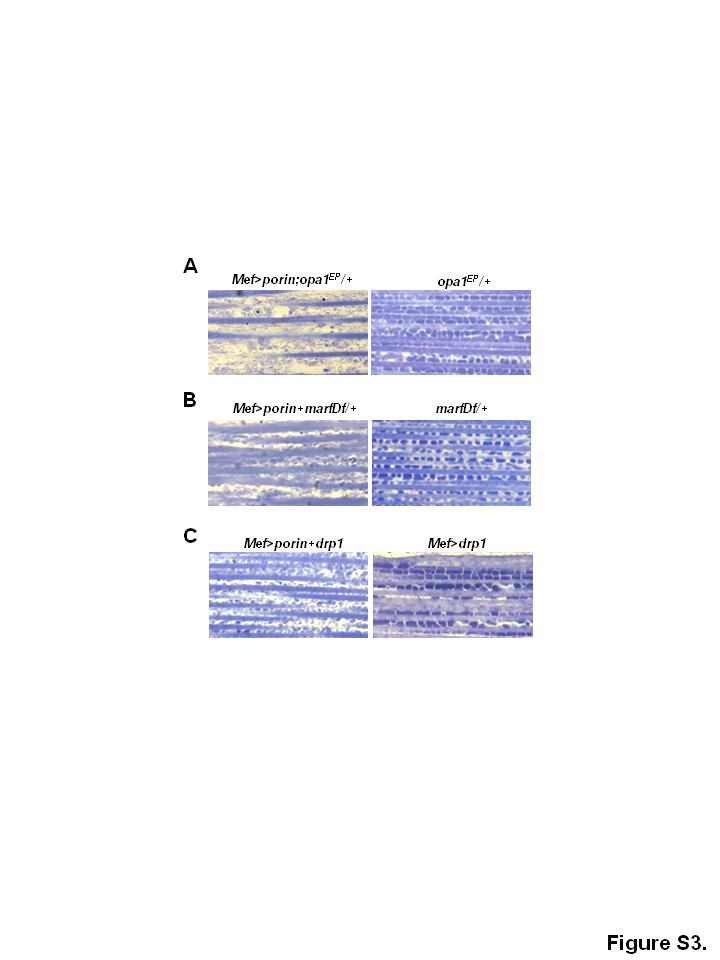

Supplement: Figure S3 — Genetic interaction analysis between Porin and the components involved in mitochondrial remodeling process. (A–C) Longitudinally sectioned thorax images stained with toluidine blue. Details of all indicated genotypes are described in Supplementary Information. (0.35 MB TIF) [file pone.0013151.s004.tif]

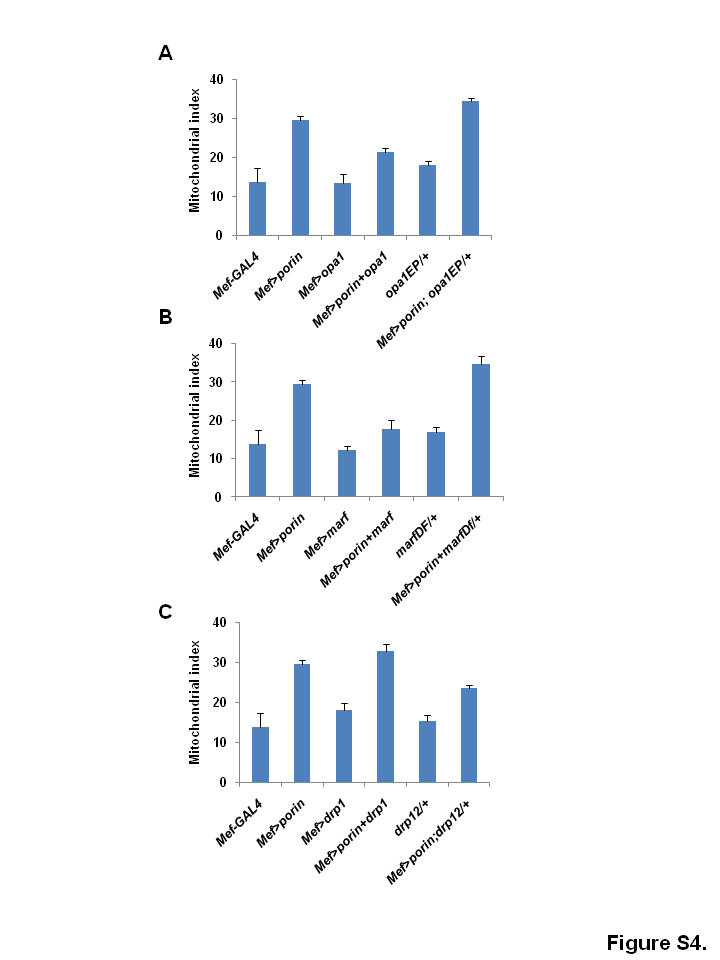

Supplement: Figure S4 — Quantification of the thorax mitochondria phenotype. (A–C) Measurement of the number of mitochondria within 50 um distance between two thorax muscle fibers (mitochondrial index) in each genotypes. Genetic interaction analysis between porin and opa1 (A), marf (B), and drp1 (C). n>10. (0.08 MB TIF) [file pone.0013151.s005.tif]
